# Comparison of analgesic effect of oxycodone and morphine on patients with moderate and advanced cancer pain: a meta-analysis

**DOI:** 10.1186/s12871-018-0583-8

**Published:** 2018-09-24

**Authors:** Kai-Kai Guo, Cheng-Qi Deng, Gui-Jun Lu, Guo-Li Zhao

**Affiliations:** 1 Department of Pain Management, The Center of Anaesthetized Operation, Chinese PLA General Hospital, No. 28 Fuxing Road, Beijing, 100853 China; 2 Department of Anesthesiology, First Affiliated Hospital of General Hospital of PLA, Beijing, 100048 China

**Keywords:** Oxycodone, Morphine, Cancer pain, Meta-analysis

## Abstract

**Background:**

Morphine and oxycodone are considered as wide-spreadly used opioids for moderate/severe cancer pain. However, debate exists about the evidence regarding their relative tolerability and underlying results.

**Methods:**

A systematic search of online electronic databases, including PubMed, Embase, Cochrane library updated on October 2017 were conducted. The meta-analysis was performed including the studies that were designed as randomized controlled trials.

**Results:**

In total, seven randomized clinical trials met our inclusion criteria. No statistical differences in analgesic effect between oxycodone and morphine were observed. Both the pooled analysis of API (MD =0.01, 95% CI -0.22 – 0.23; *p* = 0.96) and WPI (MD = − 0.05, 95% CI -0.21 – 0.30; *p* = 0.72) demonstrated clinical non-inferiority of the efficacy of morphine compared with oxycodone, respectively. Additionally, no significant difference in PRR response was observed in either oxycodone or morphine that were used in patients (MD =0.99, 95% CI -0.88 – 1.11; *p* = 0.87). With the pooled result of AEs indicating the comparable safety profiles between the 2 treatment groups, the meta-analysis on the nausea (OR = 1.20, 95% CI 0.90–1.59; *p* = 0.22), vomiting (OR = 1.33, 95% CI 0.75–2.38; *p* = 0.33), somnolence (OR = 1.35, 95% CI 0.95–1.93; *p* = 0.10), diarrhea (OR = 1.01, 95% CI 0.60–1,67; *p* = 0.98), and constipation (OR = 1.04, 95% CI 0.77–1.41; *p* = 0.79) was conducted, respectively.

**Conclusions:**

In the current study, no remarkable difference was identified either in analgesic efficacy or in tolerability of oxycodone and morphine as the first-line therapy for patients with moderate to severe cancer pain. Thus, no sufficient clinical evidence on the superior effects of oxycodone to morphine was provided in this experimental hypothesis.

## Background

The principle to treat cancer pain, the 3 step analgesic ladder, was developed by the World Health Organization (WHO) in 1986. This 3-step ladder treatment recommends the use of non-opioid analgesics for weak opioids and strong opioids for moderate–severe pain, indeed, these agents have guided the most effective treatment for cancer pain [1].

Overall there has been an increase of cancer patients attain satisfactory analgesia based on opioid therapy [2]. Opioids used to treat moderate-to-severe pain, if appropriately prescribed [3, 4]. Oral morphine has traditionally been widely used for treating patients with moderate or severe pain according to the WHO ladder Step-III [5, 6]. Reduced fluctuations in drug plasma concentrations are predicted to provide acceptable adverse effects and, potentially, better efficacy of taking short-acting opioids [7].

However, other strong opioids such as oxycodone can be used as alternatives [8, 9]. According to European Association for Palliative Care recommendations, oxycodone was alternative used in cancer patients to morphine in the 2001, and it frequently recommended in clinical use [10, 11]. Oxycodone (OX) has been showed a similar profile and has been found to provide statistically reduction in pain, primarily in the central nervous system [12].

More recent guidelines have recommended morphine and oxycodone as first-line opioids to treat cancer pain, while the evidence is limited [13, 14]. Several trails have compared the analgesic efficacy and adverse effects of different opioids conducted in cancer patients [15–17]. A comparable analgesic efficacy and safety profile was observed during the treatment, even though differences in design and assessment methods. No significant differences were found in two open-label RCTs comparing the first- line administration of morphine and oxycodone [17, 18].

Indeed, cancer patients with anxiety and depression experience an improvement in symp- toms of pain [21]. This would imply an inappropriate use of opioids for the “pain experience” and suffering [22]. Opioid abuse and addiction the most frequently reported in cancer pain patients receiving opioid analgesia [23–26]. In addition, the successful management of cancer pain is based on achieving adequate symptom relief with minimal adverse events (AEs) in a manner convenient for patients. Moreover, most RCTs comparing different opioids lack a good evidence base in the definition, measurement, and reporting of adverse events (AEs) [19].

On this basis, we launched this study aimed at assessing the effectiveness and safety of comparing morphine versus oxycodone in patients with cancer pain.

## Methods and materials

### Search strategy

Two investigators independently searched electronic databases: Pubmed, Embase, Cochrane library up to October 2017.The process was established to find all articles with the keywords: “oxycodone” “morphine” and “cancer pain”, and relevant Medical Subject Heading (MeSH) terms were utilized. The reference lists of all articles that dealt with the topic of interest were also hand-searched to check for additional relevant publications.

Medical Subject Heading (MeSH) terms including morphine, oxycodone, neoplasms,and pain.

### Eligibility criteria

Studies were included in the meta-analysis should meet the following criteria: (1) the studies are designed as randomized controlled trials; (2) the primary outcome was to compare the patients with cancer-related pain who responded clinically to morphine and oxycodone as first-line treatment; (3) studies providing data of analgesic effect for both two groups, and Hazard Ratio (HR) with corresponding 95% CIs were provided; If we found duplicated or overlapped data in multiple reports, we just include the one with most complete information.

We excluded from the current meta-analysis: (1) non-randomized controlled studies; (2) data on the main outcomes were unavailable; (3) Studies including patients receiving either morphine or oxycodone combined with other drugs for cancer pain.

### Quality assessment

Two investigators separately rated the quality of the retrieved studies. Study quality was assessed using Newcastle-Ottawa Quality Assessment Scale.

### Data extraction

Two authors independently extracted the relevant data from each trial. Disagreement was revolved by consensus. From each of the eligible studies, the main categories based on the following: first author family name, publication year, study period, country, number of patients, study interventions, analgesic effect parameters (Pain Relief Rate, Average Pain Inventory and Worst Pain Inventory), and adverse reaction parameters (nausea, vomiting, somnolence, diarrhea, and constipation).

### Statistical analysis

A sensitivity analysis was also performed to examine the impact on the overall results, depending on the heterogeneity across the included studies. Heterogeneity was examined by calculating I^2^ statistic [27]. Heterogeneity with an I^2^ of 25–50%, 50–75%, or > 75% were indicated low, moderate, or high heterogeneity, respectively [28]. When there was low heterogeneity among studies, data were analyzed using a fixed-effects model. Otherwise, the random effects model was used. *p*-value of 0.05 was considered statistically significant. The statistical analyses were per- formed using Review Manager version 5.3 software (Revman; The Cochrane collaboration Oxford, United Kingdom). The results of our meta-analysis were shown in forest plots. The Begg test and the Egger test were conducted to evaluate publication bias.

## Results

### Overview of literature search and study characteristics

**Fig. 1:**
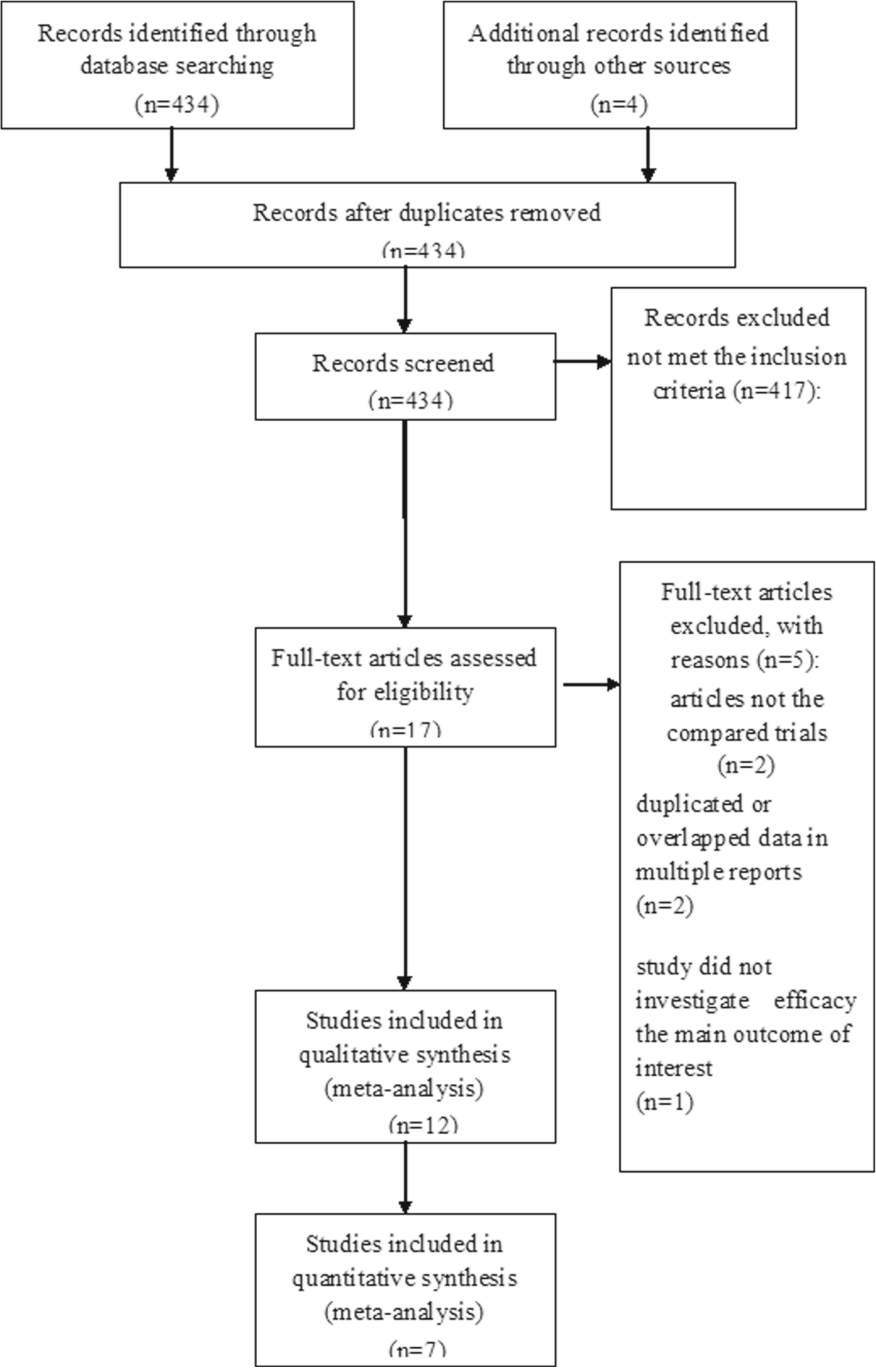
PRISMA flow chart of selection process to identify studies eligible for pooling

A total of 434 studies were retrieved initially for evaluation. Based on the criteria described in the methods, 12 publications were evaluated in more detail, but some did not provide enough detail of outcomes of two approaches. Therefore, a final total of 7 [17, 29–34] RCTs were included in this meta-analysis. The search process is described in Fig. 1.

All included studies in this study were based on moderate to high quality evidence. Table 1 describes the primary characteristics of the eligible studies in more detail.

**Table 1:** the primary characteristics of the eligible studies in more detail

Study Year	Country	Study period	Age		N		Interventions
			morphine	oxycodone;	morphine	oxycodone;	
Nashat Y 2003	USA	NR	NR	NR	20	22	over-encapsulated oxymorphone ER (10, 20, or 40 mg) or over- encapsulated oxycodone CR (20, 40, or 80 mg).
Satoshi Inoue 2017	Japan	2014–2015	70.1	68.4	86	92	hydromorphone extended-release tablets plus placebo oxycodone hydrochloride extended-release tablets 4 mg/day or placebo hydromorphone extended-release tablets plus oxycodone hydrochloride extended-release tablets 10 mg/day orally for 7 days (once-daily dosing for hydromorphone and twice-daily dosing for oxycodone)
O. Corli 2016	Italy	2011.5–2014.7	67.5	66.9	122	125	morphine or oxycodone for 28 days.The initial dose of opioid was based on the recommendations of the European Association for Palliative Care/EAPC
Julia Riley 2015	UK	2006.5–2011.7	59.2	58.9	100	100	the starting dose was determined by the treating physician on an individual patient basis and titrated accordingly until adequate pain control was achieved or intolerable side effects were re- ported by the patient.
Shiying Yu 2014	China	NR	53.5	52.7	125	123	require between 40 and 184 mg of oral morphine or morphine equivalents every 24 h for chronic management of cancer pain
S. Mercadante 2010	Italy	NR	NR	NR	21	25	receive 30 mg/d of sustained release oralmorphine or sustained release oral oxycodone(20 mg/d).
Ernesto Zecca 2016	Italy	2006.9.14–2007.12.21	61.8	62.1	95	92	Opioid dosages were reported as oral morphine equivalent daily dose (MEDD) mg, converted using a 1.5:1 ratio between morphine and oxycodone.

### Clinical and methodological heterogeneity

#### Pooled analysis of pain relief rate (PRR) comparing oxycodone with morphine on patients with cancer pain

**Fig. 2:**
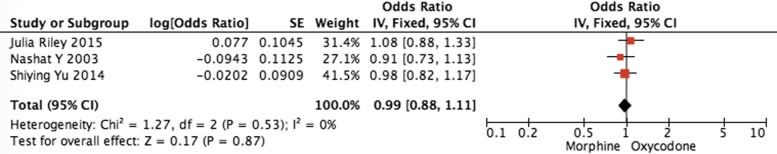
Pooled analysis of PRR comparing oxycodone with morphine on patients with cancer pain

The pooling analysis [29, 32, 33]revealed that there was no difference in composite PRR between oxycodone and morphine (MD =0.99, 95% CI -0.88 – 1.11; *p* = 0.87) (Fig. 2).

#### Pooled analysis of pain inventory (PI) comparing oxycodone with morphine on patients with cancer pain

**Fig. 3:**
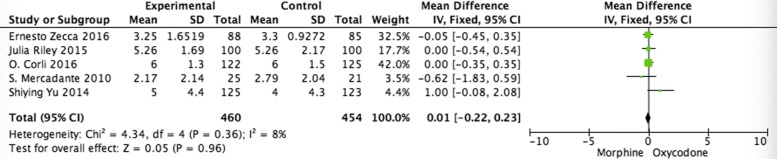
Pooled analysis of API comparing oxycodone with morphine on patients with cancer pain

**Fig. 4:**
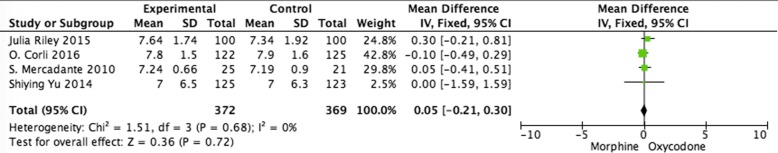
Pooled analysis of WPI comparing oxycodone with morphine on patients with cancer pain

A fixed- effects model was used to pool the PI data, since the heterogeneity across the four studies was very low. Both the pooled analysis of average pain inventory (API) (MD =0.01, 95% CI -0.22 – 0.23; *p* = 0.96) (Fig. 3) [17, 31–34] and worst pain inventory (WPI) (MD = − 0.05, 95% CI -0.21 – 0.30; *p* = 0.72) (Fig. 4) [17, 31–33] demonstrates clinical non-inferiority of the efficacy of morphine compared with oxycodone, respectively.

#### Pooled analysis of adverse reaction parameters comparing oxycodone with morphine on patients with cancer pain

**Fig. 5:**
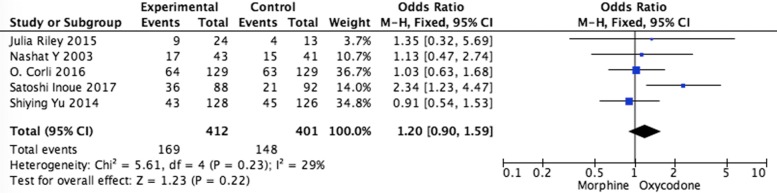
Pooled analysis of nausea comparing oxycodone with morphine on patients with cancer pain

**Fig. 6:**
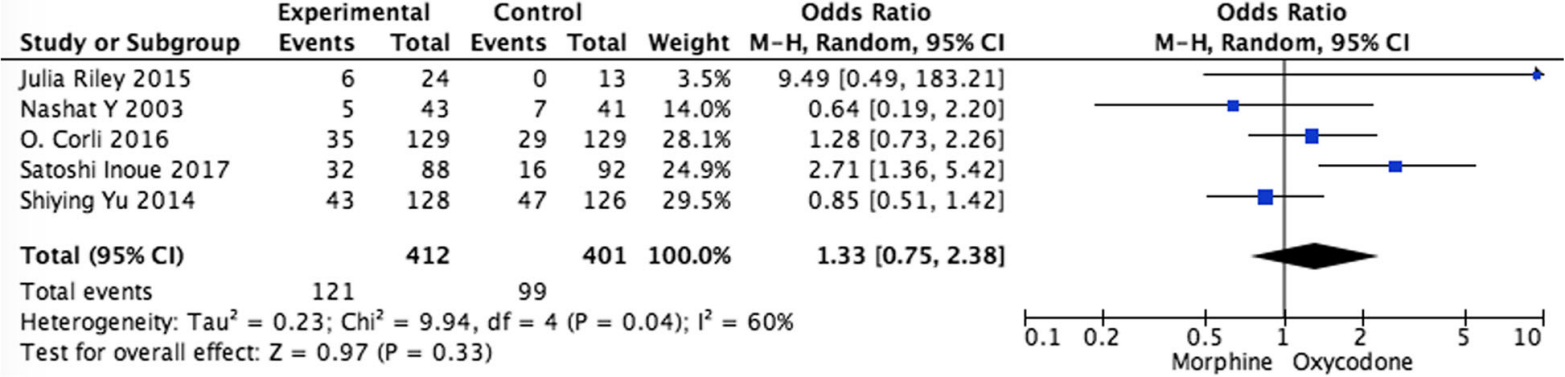
Pooled analysis of vomiting comparing oxycodone with morphine on patients with cancer pain

**Fig. 7:**
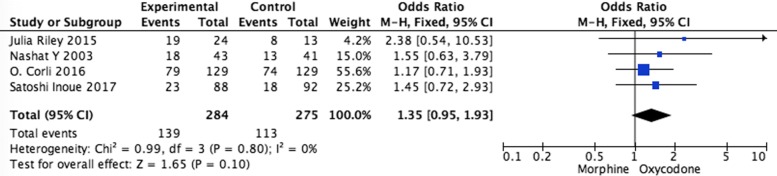
Pooled analysis of somnolence comparing oxycodone with morphine on patients with cancer pain

**Fig. 8:**
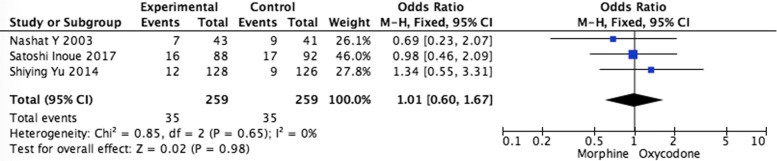
Pooled analysis of diarrhea comparing oxycodone with morphine on patients with cancer pain

**Fig. 9:**
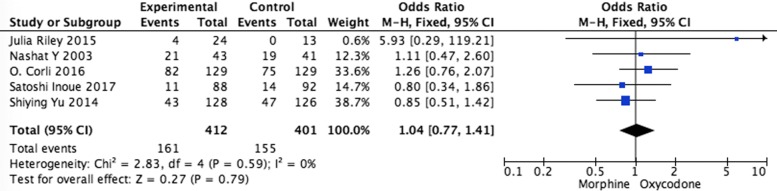
Pooled analysis of constipation comparing oxycodone with morphine on patients with cancer pain

Systematic evaluations of adverse effects (AEs) data analysis were shown in Figs. 5, 6, 7, 8 and 9. The most common treatment-related adverse events are nausea (OR = 1.20, 95% CI 0.90–1.59; *p* = 0.22), vomiting (OR = 1.33, 95% CI 0.75–2.38; *p* = 0.33), the somnolence (OR = 1.35, 95% CI 0.95–1.93; *p* = 0.10), diarrhea (OR = 1.01, 95% CI 0.60–1,67; *p* = 0.98), and constipation (OR = 1.04, 95% CI 0.77–1.41; *p* = 0.79) the difference had no statistical significance between oxycodone and morphine.

## Discussion

For patients with cancer pain, it is important to select the most appropriate manage- ment of moderate/severe cancer pain regardless of their disease stage to have a positive effect on the quality of life. Opioids are the mainstay of treatment for cancer pain at the second and third steps according to the 3-step analgesic ladder of the World Health Organization [1, 5, 35].

Morphine-like opioids are frequently used and can be interchangeable. The recently published EAPC recommendations [13] indicate that pain control did not differ significantly between morphine, oxycodone, and hydro-morphine, permitting a weak recommendation that any one of these drugs can be recognized as the treatment of choice for the treatment of moderate/severe pain in cancer patients.

The results of this meta-analysis demonstrate clinical non-inferiority of morphine compared with oxycodone in alleviating cancer pain, with respect to achieved a comparable clinical response whether morphine or oxycodone was used as first-line opioid in the treatment of cancer-related pain. The underlying opioid-related mechanisms for cancer patients including the inflammatory factors or apparent sprounting and then destruction of sensory, which are able to allow for assessment of pain in the early stage of disease [36].

Opioid metabolism was consistent with genetic differences and complex concomitant medications. The results of our analysis could be attributed to different factors.

First, at a molecular level, oxycodone has been identified as a different metabolic pathway compared with morphine. Intriguingly, some previous studies have demonstrated that morphine and oxycodone are addressed the underlying different opioid-related mechanisms: m- and k-opioid receptors, respectively [37, 38]. Oxycodone and its metabolites may have sufficient m-receptor activity [39], and an aggressive local disease, like cancer, may exclusively on peripheral k-opioid receptors. The k-opioid receptor agonists are specifically target analgesics in experimental models of visceral pain, acting peripherally [40, 41]. It has been shown that oxycodone was actually associated with benefit in the treatment of visceral pain than morphine [42, 43], it is perhaps that the prevalent peripheral activity of oxycodone as a putative k-agonist, and a relatively low affinity for m- receptors [44, 45].

Secondly, although morphine and oxycodone are widely used for cancer pain, previous report indicated that their effects on immune system are different. Chronic morphine treatment induces immunosuppression and abnormality of some immunological indexes including interleukin 2 production and natural killer (NK) cell activity. These findings emerged in an exploratory prospective study in animals and in vitro [46–48].

Other studies have indicated relationship between morphine and the susceptibility to infections in animals [49, 50]. On the other hand, prospective study showed no relationship between oxycodone and the immunological indexes [48]. Moreover, compared to morphine itself, morphine’s metabolites (morphine-3- glucuronide and morphine-6-glucuronide) have similar effects on several immunological indexes [51, 52]. Based on the above pharmacological property background, morphine might be more effective than oxycodone. It is known that immunosuppression has effect on the development of infections, disease progression, and the choice of cancer therapy. Therefore, in the selection of opioids, immune system should be considered in cancer patients with pain.

Thirdly, the efficacy of different individual gene polymorphism on the pharmacodynamic effects of opioids in painful conditions is controversial [53–55]. The efficacy of various opioids in patients with cancer based on different receptors and their subtypes [55]. Genetic differences in the expression of different receptor subtypes could affect individual therapeutic efficacy [55–59].While, these results have not been replicated in different populations, because of differences in definition of phenotype and outcome measures used [60].

With regard to therapeutic safety, although opioids are the first-line drug for moderate to severe cancer pain [3, 4], opioid-related AEs (particularly at high doses) that led to discontinuation of study treatment can sometimes prevent adequate analgesia [20]. Heiskanen and Kalso [61] have shown similar results of adverse effects with the two drugs, reporting vomiting was more common (*p* < 0.01) with morphine, while constipation more frequent with oxycodone (*p* < 0.01). Mucci Lo Russo et al. [62] showed adverse effects with a statistically significant lower incidence regarding itching with oxycodone (*p* < 0.04) and two cases of hallucinations with morphine. While, other authors did not found significant differences in adverse effects [17, 18, 63].

Several questions remain to be answered. First, although some preclinical data are available in cancer pain models, the mechanism of action of morphine and oxycodone for moderate-to-severe pain remains to be debated. Second, different subgroups of patients might have differential responses to cancer pain treatment. Future research should aim to identify these subgroups, probably through the identification and validation of biomarkers, to refine the population of patients likely to obtain benefit from morphine and oxycodone.

## Conclusion

In conclusion, morphine showed no inferiority or superiority when compared with oxycodone in terms of analgesic response or adverse effects. Currently, growing evidence has suggested that the pharmacokinetics and pharmacodynamics are different among opioids. As a result, different opioids are likely to differ in some respects. Additional studies, including comparison with controls and other opioids, will be necessary to confirm the results. Moreover, in the future, prospective, randomized, clinical trials are designed to evaluate the efficacy of different opioids in patients with cancer pain based on gene polymorphism.

## Abbreviations

AEs: Adverse events
API: Average Pain Inventory
CROM: Morphine
CROO: Oxycodone
HR: Hazard ratio
MD: Mean difference
OR: Odds ratio
PRR: Pain Relief Rate
WHO: World Health Organization
WPI: Worst Pain Inventory
