# Correction to: Comparison of analgesic effect of oxycodone and morphine on patients with moderate and advanced cancer pain: a meta-analysis

**DOI:** 10.1186/s12871-018-0630-5

**Published:** 2018-11-19

**Authors:** Kai-Kai Guo, Cheng-Qi Deng, Gui-Jun Lu, Guo-Li Zhao

**Affiliations:** 1 Department of Pain Management, The Center of Anaesthetized Operation, Chinese PLA General Hospital, No. 28 Fuxing Road, Beijing, 100853 China; 2 Department of Anesthesiology, First Affiliated Hospital of General Hospital of PLA, Beijing, 100048 China

## Correction

After publication of this article [1], the authors noted that the corresponding email address is incorrect. The correct correspondence address is luguijun301@163.com. The correct correspondence address can also be seen below.
